# Transthyretin Cardiac Amyloidosis and Heart Failure: State-of-the-Art Review and Practice Guidance

**DOI:** 10.31083/RCM46181

**Published:** 2025-12-24

**Authors:** Syed Bukhari, Mohammad Hamza, Aslam Malik

**Affiliations:** ^1^Division of Cardiology, Department of Medicine, Johns Hopkins University, Baltimore, MD 21218, USA; ^2^Department of Internal Medicine, TidalHealth, MD 21801, USA; ^3^Department of Internal Medicine, St. Luke's Healthcare, Sellersville, PA 18960, USA

**Keywords:** transthyretin cardiac amyloidosis, heart failure, heart failure with reduced ejection fraction, guideline-directed medical therapies, amyloid-specific therapies

## Abstract

Transthyretin cardiac amyloidosis (ATTR-CA) is an increasingly recognized and underdiagnosed cause of heart failure (HF), encompassing both preserved (HFpEF) and reduced (HFrEF) ejection fraction phenotypes. Once identifiable only following a biopsy, the advent of bone scintigraphy has dramatically improved noninvasive detection and detected a higher community prevalence, particularly among older patients with unexplained left ventricular hypertrophy. ATTR-CA arises from misfolding of transthyretin (TTR), leading to amyloid fibril deposition within the myocardium, which impairs cardiac compliance, conduction, and output. This review explores the evolving epidemiology of ATTR-CA in HF, mechanisms of disease progression, and key features for screening, emphasizing clinical red flags, biomarkers, and imaging features. This review also addresses the nuanced role of guideline-directed medical therapy in this population, where neurohormonal agents may offer limited benefit or be poorly tolerated due to restrictive physiology and autonomic dysfunction. Crucially, the emergence of amyloid-specific therapies, including TTR silencers, stabilizers, and degraders, has transformed the therapeutic landscape, offering mortality and morbidity benefits that were previously unavailable. Early diagnosis and individualized management, integrating conventional and amyloid-targeted approaches, are essential to improving outcomes in this complex and increasingly treatable cardiomyopathy.

## 1. Introduction

Transthyretin cardiac amyloidosis (ATTR-CA) has increasingly been recognized as an important etiology of heart failure (HF), driven in part by the advent of bone-avid, technetium-labeled pyrophosphate tracers specific to ATTR-CA [1]. These imaging agents have revealed an unexpectedly high community prevalence of a disease that was previously diagnosed only through endomyocardial biopsy [2].

Once thought to be associated almost exclusively with HF with preserved ejection fraction (HFpEF), recent data demonstrate that a substantial proportion of patients with ATTR-CA present with HF with reduced ejection fraction (HFrEF) at the time of diagnosis [3]. As a clinical mimic of HFpEF, timely diagnosis of ATTR-CA is critical—particularly given the emergence of amyloid-specific therapeutics that offer morbidity and mortality benefits in this unique patient population.

This review will explore the evolving epidemiologic paradigm of ATTR-CA in HF, the pathophysiologic mechanisms leading to both HFpEF and HFrEF phenotypes, and the current evidence on the use of guideline-directed medical therapies (GDMT) in patients with ATTR-CA, including mechanistic explanations for their limited tolerability in this setting. We will also examine the data for amyloid-specific therapies, focusing on strategies to reduce transthyretin production, stabilize the native protein, and explore the ongoing efforts to promote resorption of existing amyloid fibrils.

## 2. Pathogenesis of ATTR-CA

**Fig. 1.**
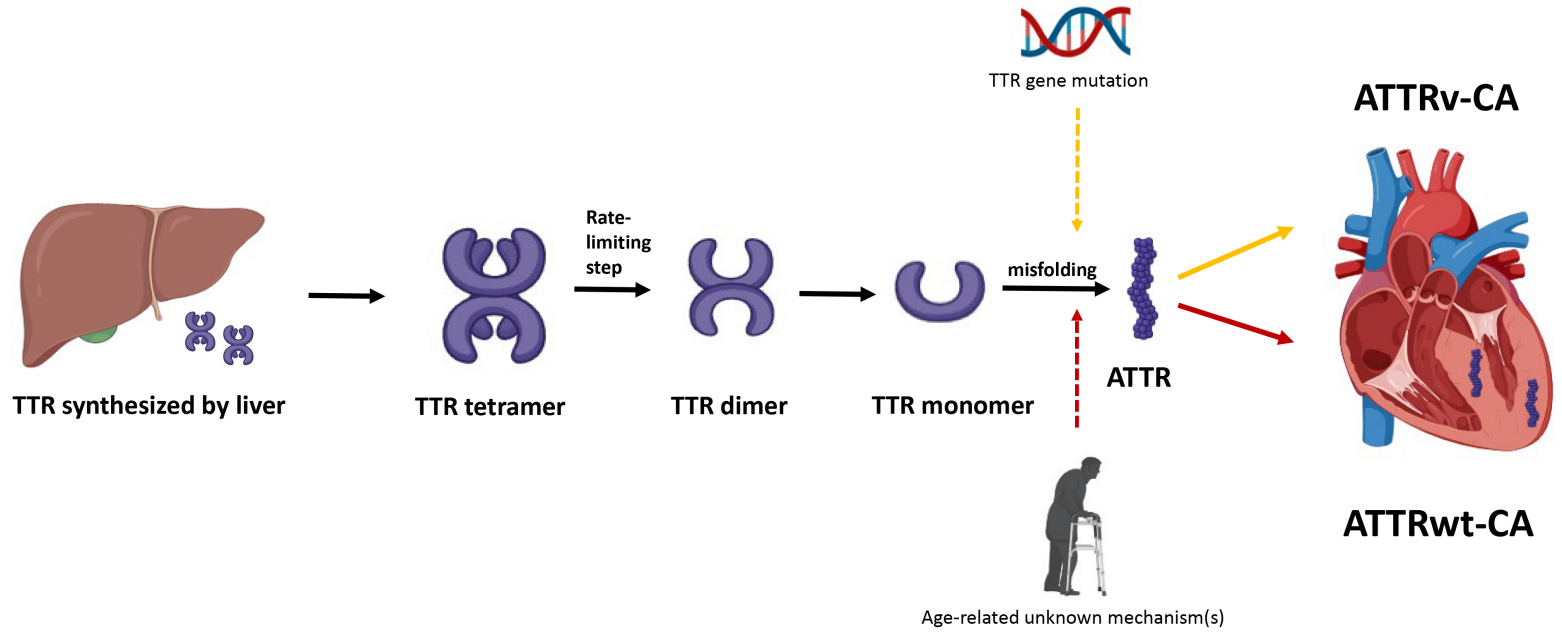
**Pathogenesis of transthyretin cardiac amyloidosis**. Hepatically-derived TTR undergoes misfolding, either due to mutation in *TTR* gene or secondary to age-related unknown mechanisms, and the resulting ATTR is subsequently deposited in the myocardial interstitium. TTR, transthyretin; ATTR, transthyretin amyloid fibril; ATTRv-CA, variant or hereditary transthyretin cardiac amyloidosis; ATTRwt-CA, wild-type transthyretin cardiac amyloidosis.

The pathogenesis of ATTR-CA centers on the destabilization and misfolding of the transthyretin (TTR) protein, a tetramer primarily synthesized in the liver that transports thyroxine and retinol (vitamin A) in the plasma (Fig. 1). ATTR-CA is divided into 2 subtypes. Hereditary or variant ATTR-CA (ATTRv-CA) arises from mutations in the *TTR* gene, with over 130 mutations identified to date, which weaken the tetramer’s structural integrity. In wild-type ATTR-CA (ATTRwt-CA), age-related biochemical changes—though not fully understood—similarly reduce tetramer stability. The tetramer dissociates into monomer, which subsequently misfolds into amyloid fibrils. These fibrils deposit in tissues, including the heart, leading to progressive organ dysfunction. The degree of cardiac involvement at the time of diagnosis is the most important prognostic indicator.

## 3. Epidemiology of ATTR-CA in HF

### 3.1 Prevalence in HFpEF

ATTR-CA is now increasingly recognized as a significant and underdiagnosed contributor to HFpEF, though its true prevalence remains elusive. Estimates range widely—from as low as 3% to as high as 20% —largely due to methodological variability across studies [4, 5, 6, 7, 8]. These differences include patient selection criteria, age cutoffs, left ventricular (LV) wall thickness thresholds, and the degree of diagnostic scrutiny.

A recent Spanish study comprising 387 older individuals with HFpEF and LV hypertrophy (LVH, defined as wall thickness ≥12 mm) reported the prevalence of ATTR-CA to be 16.8% [4]. This finding is consistent with a prior multicenter investigation from Spain that included over 400 older patients aged ≥65 years with LVH, in which the prevalence was 20% [5]. A Swedish cohort that investigated 134 patients with HF and an LV wall thickness >14 mm also reported prevalence of ATTR-CA in one in five patients (20%) [8]. These data underscore that among elderly patients with HF and unexplained LVH, ATTR-CA is relatively common and may be underdiagnosed. In contrast, a study conducted in the Netherlands, which evaluated 252 patients with unselected HFpEF found a markedly lower prevalence of ATTR-CA at just 3% [6]. Notably, one-third of the ATTR-CA patients had normal LV thickness in this cohort, representing an earlier disease stage. Hence, depending solely on LV wall thickness in diagnosing ATTRCA can result in delayed or overlooked diagnoses. These findings collectively suggest that targeted screening based on age and LV wall thickness may be necessary to identify ATTR-CA more effectively within HFpEF populations.

The prevalence of ATTRwt-CA and ATTRv-CA is also markedly different. ATTRwt-CA is a predominant subtype in elderly patients with HF. In a prospective study evaluating 120 patients with HFpEF, the prevalence of ATTRwt-CA, defined as positive nuclear scintigraphy and absence of *TTR* gene mutations, was found to be 13% [9]. On the other hand, ATTRv-CA is seen among certain mutations that have predilection to cardiac involvement. ATTRv-CA is a progressive disease affecting multiple systems, with clinical manifestations that vary from mainly polyneuropathy to predominant cardiomyopathy [10]. One of the important variants is V122I ATTRv-CA, which is present almost exclusively in Afro-Caribbean population in the United States, and predominantly involves cardiomyopathy [11]. Another mutation is T60A ATTRv-CA, which is predominantly seen in individuals of Irish descent [12]. According to the Irish Amyloidosis Network, the clinical presentation of T60A ATTRv-CA in Ireland usually involves individuals developing symptoms in their seventies, with neuropathy emerging as the initial and predominant feature, preceding the development of HF–driven cardiac involvement [13].

To summarize, determining the true prevalence of ATTR-CA in HFpEF remains challenging. Most studies focus on patients with clear phenotypic signs, often missing early disease in those without left ventricular thickening or typical red flags. As a result, the overall burden is likely underestimated, and opportunities for early, disease-modifying treatment are missed. Broader, systematic screening using sensitive tools—such as bone scintigraphy, advanced imaging, and circulating biomarkers—is essential to detect subclinical cases, better understand disease progression, and enable timely intervention.

### 3.2 Prevalence in HFrEF

ATTR-CA, once considered a condition confined to HFpEF, is now increasingly recognized in patients with HFrEF. Approximately one-third of patients present with HFrEF at the time of ATTR-CA diagnosis, reflecting ‘burnt-out’ or advanced disease and portending a worse prognosis compared with those who have HFpEF [14]. However, the prevalence of ATTR-CA exclusively among patients with HFrEF remains underexplored. In a meta-analysis of 11 studies including 3303 patients with HF, the pooled prevalence of ATTR-CA in HFrEF was estimated at 11.3%, derived from only two studies, compared with pooled prevalence of 15% in HFpEF [15]. In a separate study of 75 patients with unexplained HF and systolic LV dysfunction, the prevalence of wild-type ATTR-CA was approximately 9% [16]. These findings underscore the need for larger, dedicated studies to more accurately define the burden of ATTR-CA in HFrEF, which in turn will help refine community-based prevalence estimates of ATTR-CA.

## 4. Mechanisms of HF in ATTR-CA

ATTR-CA is characterized by the extracellular deposition of ATTR as insoluble amyloid fibrils within the myocardium, leading to progressive myocardial stiffening [17]. The loss of myocardial compliance increases LV filling pressures and impairs ventricular relaxation, a hallmark feature of early-stage disease. As the disease progresses, amyloid accumulation also affects the conduction system, coronary microvasculature, and valves, exacerbating cardiac dysfunction. Amyloid deposition in the atria and conduction pathways can lead to arrhythmias such as atrial fibrillation [18] and various degrees of heart block [19]. Microvascular dysfunction due to amyloid infiltration impairs coronary perfusion, contributing to subendocardial ischemia and myocyte death despite unobstructed epicardial coronary arteries. The progressive myocardial infiltration increases wall thickness without true hypertrophy, leading to a pseudo-hypertrophic appearance and further compromising cardiac output. In later stages, systolic function may deteriorate, resulting in overt HFrEF.

Furthermore, the pathophysiological cascade in ATTR-CA involves neurohormonal activation secondary to decreased cardiac output. This compensatory mechanism—driven by activation of the renin-angiotensin-aldosterone system (RAAS) and sympathetic nervous system—exacerbates fluid retention, vasoconstriction, and ventricular remodeling. Combined with the fixed stroke volume imposed by the stiffened ventricle, these processes culminate in the classic clinical syndrome of HFpEF, progressing in many cases to advanced, treatment-refractory HF. The infiltrative process is particularly insidious in ATTRwt-CA, which typically presents in older individuals with subtle symptoms and is often misdiagnosed as hypertensive heart disease or hypertrophic cardiomyopathy (HCM).

## 5. Screening for ATTR-CA in HF

### 5.1 Extracardiac Features

**Fig. 2.**
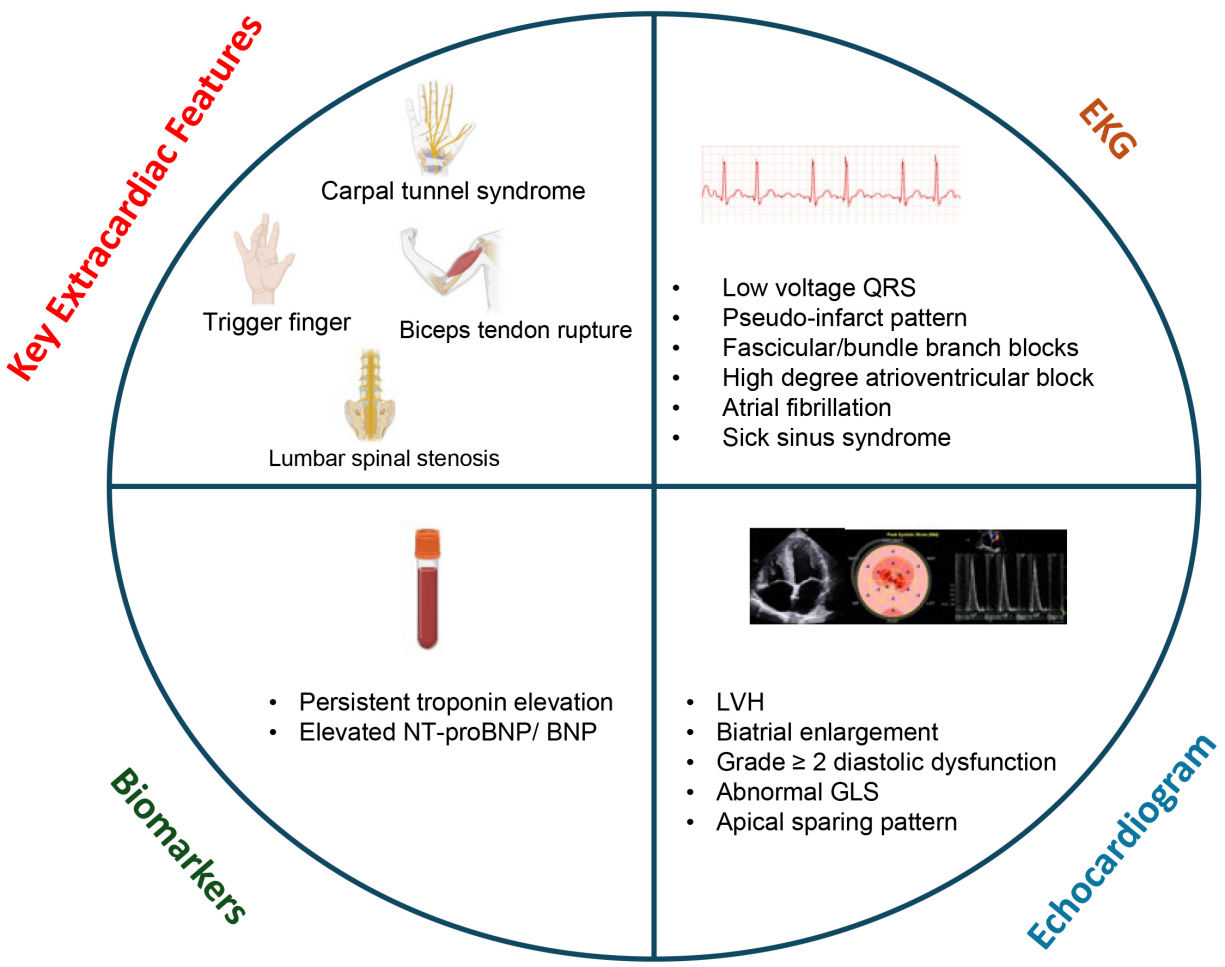
**Screening transthyretin cardiac amyloidosis in heart failure**. These red flag features on clinical exam, laboratory evaluation, EKG and echocardiography may raise suspicion for cardiac amyloidosis. GLS, global longitudinal strain; LVH, left ventricular hypertrophy; EKG, electrocardiogram; NT-proBNP, N-terminal pro-brain natriuretic peptide.

While ATTR-CA and HF, particularly HFpEF, have some demographic, clinical and echocardiographic commonalities, there are certain clues on clinical history, laboratory workup, electrocardiogram (EKG) and echocardiography that can raise initial suspicion for ATTR-CA [20] (Fig. 2). Patients with ATTR-CA, more specifically ATTRwt-CA, often have extracardiac features that are predominantly musculoskeletal, resulting from ATTR deposition in the ligaments and tendons. Carpal tunnel syndrome is probably the most common musculoskeletal manifestation that has a prevalence of up to 50% in patients with ATTR-CA and is known to precede the onset of cardiac dysfunction by 5–10 years in patients with ATTR-CA [21]. Roughly 10% of patients undergoing idiopathic carpal tunnel release surgery have biopsy-confirmed amyloid deposits in the tenosynovial sheath [22]. Trigger finger, which involves the same tendon sheath as carpal tunnel syndrome, is also commonly reported in patients with ATTR-CA, and biopsy during trigger finger release surgery has demonstrated a 2% yield for ATTR-CA [23]. Spontaneous biceps tendon rupture is another musculoskeletal manifestation of ATTR-CA, and a single center reported prevalence of 33% in patients with ATTRwt-CA [24]. Finally, about one-third of patients aged ≥50 years undergoing lumbar spinal stenosis surgery have ATTR deposits in vertebral ligament tissue [25, 26].

### 5.2 Cardiac Biomarkers

#### 5.2.1 Troponin

Cardiac troponin is the preferred biomarker for identifying myocardial injury and is commonly elevated in patients with ATTR-CA. Numerous mechanisms have been suggested to explain myocardial injury in ATTR-CA, including direct cytotoxicity of amyloid precursors, interstitial infiltration of amyloid fibrils, coronary microvascular dysfunction, concomitant coronary artery disease, diastolic ± systolic dysfunction, and HF. While the diagnostic value of troponin has been less extensively investigated, chronically elevated troponin is a useful screening tool for ATTR-CA, especially when taken in the context of other red flag features [27].

Persistent elevation of cardiac troponin levels may have prognostic value for ATTR-CA. Troponin is used to risk-stratify patients and predict mortality in both ATTRwt-CA and ATTRv-CA. Higher baseline troponin levels are associated with worse outcomes. Along with NT-proBNP, troponin forms the basis for Mayo staging system that predicts survival [28]. However, there are certain limitations with the use of troponin as a prognostic tool in HF. The lack of standardization in measuring troponin levels remains a challenge, as various generations of assays from different manufacturers have introduced variability, leading individual centers to favor specific assays [29]. Moreover, the relationship between variations in absolute troponin levels and corresponding changes in disease progression or status has not been established. Expert consensus suggests a 30% relative increase, using a high-sensitivity assay, as a better indication of ATTR-CA progression rather than a pre-specified absolute level, but this needs to be examined using robust data [30]. Finally, troponin levels are also influenced by kidney function due to impaired excretion [31], and since patients with ATTR-CA have varying degrees of kidney dysfunction, a cautious interpretation of variation in troponin levels is warranted.

#### 5.2.2 NT-proBNP

NT-proBNP is the gold standard biomarker in HF used in routine clinical practice [32]. While NT-proBNP does not provide diagnostic utility specific to ATTR-CA, it is an important biomarker for prognostication. NT-proBNP levels are important markers for tracking disease progression, and have been incorporated in various amyloidosis staging systems [28]. In a study of 869 UK patients with ATTR-CA (553 ATTRwt-CA, 316 ATTRv-CA), a three-stage system based on NT-proBNP and estimated glomerular filtration rate (eGFR) thresholds (Stage I: NT-proBNP ≤3000 ng/L, eGFR ≥45 mL/min; Stage III: NT-proBNP >3000 ng/L, eGFR <45 mL/min; Stage II: remainder) effectively stratified prognosis—with median survival of 69.2, 46.7, and 24.1 months for Stages I–III respectively (*p* < 0.0001); these findings remained robust after age adjustment, across genotypes, and in a 318-patient French validation cohort [33]. BNP may serve as an alternative to NT-proBNP, but has been studied less extensively, and more importantly both these markers circulate at different concentrations [34]. Therefore, when extrapolating results, careful interpretation is needed. Furthermore, care must be taken when interpreting NT-proBNP values, as elevated levels can also result from conditions such as renal dysfunction and atrial fibrillation, potentially complicating their use in monitoring ATTR-CA progression.

#### 5.2.3 EKG

Low voltage on EKG is a key finding in ATTR-CA and, while not specific, its presence in the setting of marked LVH should raise suspicion for the disease. Unlike true hypertrophy, ATTR-CA involves pseudo-LVH due to non-conducting amyloid fibril deposition and extracellular expansion, leading to disproportionately low QRS voltages on EKG despite increased myocardial wall thickness [35, 36]. In addition, ATTR-CA can be characterized by a ‘pseudo-infarct’ pattern, which refers to the presence of Q waves or QS complexes on the EKG that mimic previous myocardial infarction, typically in the anterior or inferior leads, but without corresponding evidence of coronary artery disease [37]. This is reflective of delayed conduction in amyloid-infiltrated myocardial tissue rather than ischemic injury, and can have diagnostic utility when seen together with low-voltage QRS and LVH.

EKG changes may also help in the assessment of disease progression. The development of advanced atrioventricular (AV) block and PR interval prolongation could signify disease progression, and could appear in isolation or together with bundle branch block patterns. Furthermore, the need for pacemaker implantation for bradyarrythmias (atrial fibrillation with slowed ventricular response, sick sinus syndrome and high-degree AV block) is also suggestive of progressive amyloid burden.

### 5.3 Echocardiogram

**Fig. 3.**
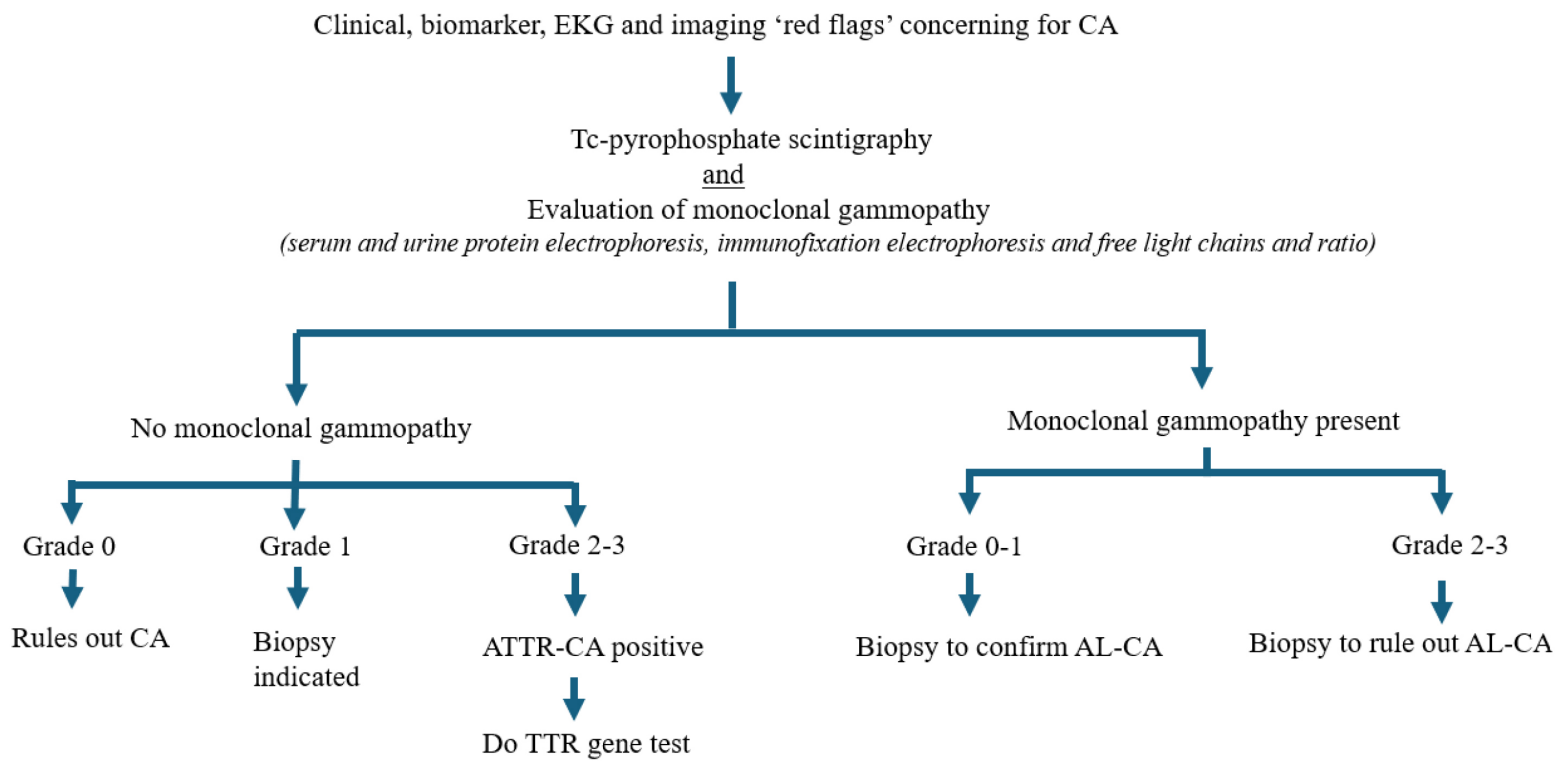
**Algorithmic flowchart demonstrating screening and diagnosis of cardiac amyloidosis**. Red flag features prompt nuclear scintigraphy, which is always performed in conjunction with serum evaluation of monoclonal gammopathy to rule out light chain amyloidosis. CA, cardiac amyloidosis; AL-CA, light chain cardiac amyloidosis; ATTR-CA, transthyretin cardiac amyloidosis; TTR, transthyretin; EKG, electrocardiogram.

ATTR-CA and garden-variety HFpEF have some overlapping features on echocardiogram. While LVH can be seen in non-amyloid HFpEF, LV thickening in ATTR-CA is significantly greater [4, 38], and is often symmetric [39]. However, it is important to note that the absence of LVH does not rule out ATTR-CA, as upto 10% of ATTR-CA patients may have normal wall thickness, particularly in the early stages of the disease [40]. Atrial enlargement, often bilateral, is a common feature in ATTR-CA together with near-normal sized ventricles, which represents increased LV preload secondary to increased myocardial stiffness—a hallmark of restrictive physiology. Advanced diastolic dysfunction (grade ≥2) is characteristically seen [41]. Severely reduced S’ and E’ velocities, which also reflect underlying restrictive and infiltrative nature of the disease, can help distinguish ATTR-CA from hypertensive heart disease and HCM. Granular sparkling myocardial appearance can be seen but is not specific for ATTR-CA. Finally, abnormal longitudinal strain with apical sparing pattern (also known as cherry-on-top appearance) is an important finding that should prompt further investigation for ATTR-CA in the appropriate clinical context (Fig. 3) [42].

## 6. Management of ATTR-CA in HF

**Fig. 4.**
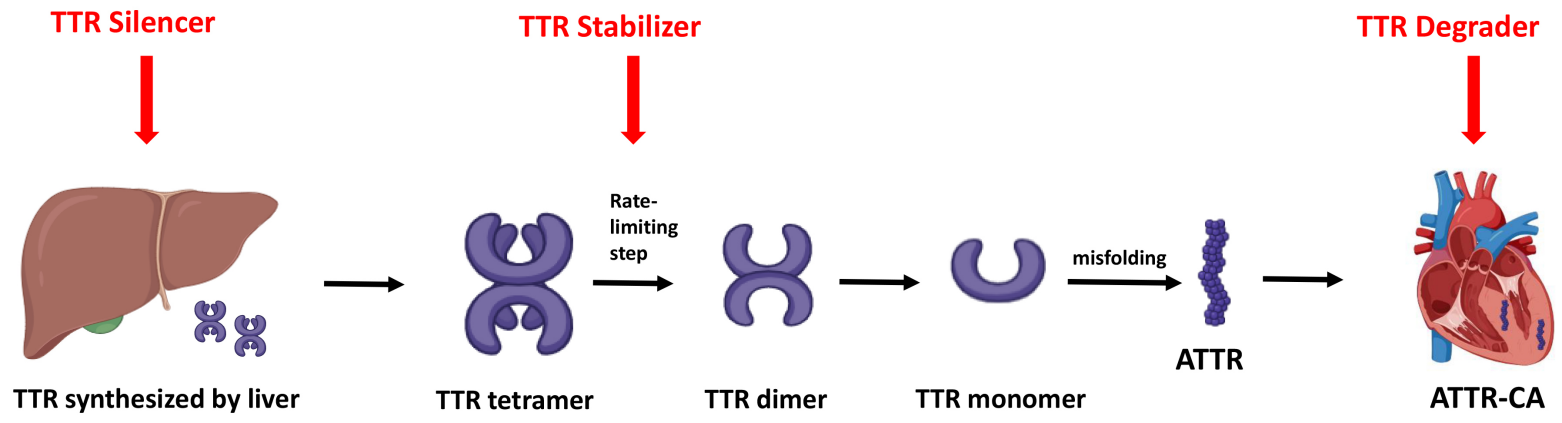
**Mechanisms of amyloid-specific therapies**. Overview of the three main therapeutic strategies for ATTR-CA: gene silencers reduce hepatic TTR production, stabilizers prevent tetramer dissociation, and degraders promote clearance of misfolded or deposited amyloid fibrils. TTR, transthyretin; ATTR, transthyretin amyloid fibril; ATTR-CA, transthyretin cardiac amyloidosis.

The treatment of ATTR-CA in HF involves a two-pronged approach. Firstly, mitigation of congestion, initiation of GDMT, and management of any co-existing arrhythmia. Secondly, initiation of amyloid-specific treatments aimed at directly stabilizing transthyretin, and/or inhibiting fibril formation (Fig. 4).

### 6.1 Conventional HF Therapies

#### 6.1.1 Diuretics

Fluid retention is a major cause of symptoms, poor quality of life, and adverse outcomes in patients with ATTR-CA and HF. Loop diuretics are mainstay therapy for symptomatic relief, helping to mitigate congestion and maintain adequate preload. While furosemide is the most commonly used agent, torsemide and bumetanide have higher potency and bioavailability [43]. The choice of a loop diuretic should be individualized and tailored to the severity of symptoms, cost and patient preferences. It is worth noting that the cardiac output in ATTR-CA is dependent on higher filling pressures given the restrictive physiology [44], thereby increasing the risk of organ hypoperfusion with aggressive diuresis. Therefore, cautious diuresis is crucial. Serum creatinine and estimated glomerular filtration rate should be closely monitored while diuresis is instituted, especially with intravenous medications while inpatient. In cases of severe fluid retention unresponsive to loop diuretics alone, metolazone can be employed intermittently at doses of 5 mg or 10 mg [45]. Metolazone is a thiazide-like diuretic that primarily inhibits sodium reabsorption in the distal convoluted tubule, thereby enhancing diuresis when used synergistically with loop diuretics. Adjunctive therapy with spironolactone, a mineralocorticoid receptor antagonist, is another potential strategy. In addition to providing a diuretic effect, spironolactone offers the benefit of mitigating hypokalemia—a common electrolyte disturbance associated with loop diuretics.

Diuretic use also carries prognostic significance. Diuretic dose and New York Heart Association (NYHA) functional class have been shown to independently predict mortality in ATTR-CA and, when incorporated into existing staging systems Mayo and UK risk scores [28, 33], significantly improve their prognostic accuracy [46]. In a study comprising 309 consecutive ATTR-CA patients, higher diuretic dose at diagnosis (per 1 mg/kg increase) was independently associated with increased all-cause mortality (adjusted HR 1.43, 95% CI 1.06–1.93) [46]. Incorporating diuretic dose and NYHA class into existing Mayo and UK risk scores improved prognostic discrimination (area under the curve (AUC) up to ~0.80) while maintaining calibration.

#### 6.1.2 GDMT

Patients with ATTR-CA were excluded from large-scale HF trials that formed the basis of current GDMT pillars in HFrEF [47, 48, 49, 50, 51, 52, 53, 54, 55], and therefore it is unknown whether conventional HF medications that have substantial benefits in patients with non-amyloid HF may also benefit in those with ATTR-CA. Patients with ATTR-CA appear to respond differently to neurohormonal (NH) blockade with angiotensin-converting enzyme inhibitors (ACEi)/angiotensin receptor blockers (ARBs), beta-blockers, and mineralocorticoid receptor antagonists (MRAs), as compared with other patients with HF. This is thought to result from poor hemodynamic tolerance due to an altered pressure–volume relationship, where stroke volume is relatively fixed and ventricular–vascular coupling may be impaired. Evidence from small studies has been inconsistent—some indicate that low doses of these medications are generally well tolerated, while others suggest they are poorly tolerated and may even lead to worse outcomes [56, 57]. The absence of large-scale clinical trials contributes to this ongoing knowledge gap. Consequently, multiple consensus guidelines recommend avoiding the use of β-blockers and ACEi/ARBs, with the European Society of Cardiology (ESC) position statement specifically advising that β-blockers be withdrawn and ACEi/ARBs avoided in all patients [58]. However, it is important to note that approximately one-third patients in the ATTR-ACT trial were receiving β-blockers or ACEi/ARBs [59]. In addition, there is some emerging data from large-scale observational studies that have provided some key insights into using these medications in ATTR-CA.

Betablockers are one of the important pillars of GDMT. In ATTR-CA, patients may experience intolerance to betablockers because of the restrictive nature of the disease, whereby they have a low, fixed stroke volume and therefore rely on relatively higher heart rates to maintain adequate cardiac output. A reduction of heart rate may negatively impair cardiac output, as well as potentially blunt the chronotropic response needed to augment cardiac output during exercise. Exacerbation of pre-existing orthostatic hypotension, worsening fatigue and, occasionally, syncope due to bradyarrhythmia can be seen in ATTR-CA with the institution of betablockers. While discontinuation of beta-blockers is seen in up to one-third patients, majority are able to continue, and few may require dose reduction. A recent large-scale observational study comprising 2371 patients with ATTR-CA who were followed up on GDMT for 28 months found use of beta-blockers to be associated with lower mortality among patients with LVEF ≤40% [HR 0.61 (95% CI 0.45–0.83), *p* = 0.002], and only 22% of patients had to discontinue beta-blockers during this period [60]. Based on our clinical experience and existing data, we recommend that patients with ATTR-CA with systolic dysfunction may be started on betablockers unless there is any contraindication and should be closely monitored for any adverse side effects. If patients develop any adverse effects, dose reduction may be considered. A low threshold for discontinuation should be maintained if symptoms persist. For ATTR-CA with preserved ejection fraction (EF), medications other than beta-blockers may be preferentially considered for blood pressure control.

ACEi/ARBs/angiotensin receptor-neprilysin inhibitor (ARNi) have traditionally been believed to be poorly tolerated in ATTR-CA. In the setting of overt restrictive physiology, afterload reduction may predispose to hypotension without improving stroke volume. ACEi/ARB/ARNi can potentially exacerbate hypotension due to amyloid-associated autonomic dysfunction in ATTR-CA. In addition, some patients with ATTR-CA may not be suitable candidates for ACEi/ARB/ARNi due to pre-existing advanced kidney disease, which is not uncommon in ATTR-CA and may or may not be directly related to the disease itself. Observational studies have shown that ACEi/ARBs do not appear to confer a mortality benefit in ATTR-CA [60], although more robust studies are needed to confirm this. However, existing data also suggest that, just like beta-blockers, ACEi/ARBs are not as poorly tolerated as previously thought, and about two-thirds of patients with ATTR-CA tend to tolerate them once initiated. Data on ARNi remain very limited. We recommend the use of ACEi/ARBs in patients with ATTR-CA in the absence of hypotension or worsening kidney function. Careful monitoring of blood pressure, kidney function, and potassium levels is required.

MRAs are thought to be third pillar of conventional HFrEF therapies. Observational studies have suggested a potential survival benefit from MRAs in ATTR-CA. In a large-scale observational study from the UK National Amyloidosis Centre, involving over 2300 patients with ATTR-CA, MRAs demonstrated favorable tolerability and potential survival benefit [60]. After a median follow-up of approximately 28 months, MRA therapy was discontinued in only 7.5% of patients, compared with discontinuation rates of 21.7% for β-blockers and 32.9% for ACE inhibitors or ARBs. In propensity score–matched analysis, MRA use was independently associated with a significantly reduced risk of mortality in the overall population (HR 0.77; 95% CI: 0.66–0.89; *p* < 0.001), as well as in the subgroup with preserved left ventricular ejection fraction (LVEF >40%) (HR 0.75; 95% CI: 0.63–0.90; *p* = 0.002) [60]. It is important to obtain baseline laboratory tests, including serum potassium and renal function, prior to initiating MRA therapy, with periodic monitoring thereafter.

Sodium-glucose cotransporter 2 inhibitors (SGLT2is) have been shown to improve the outcome of patients with HF, but patients with ATTR-CA have been excluded from all phase III trials on empagliflozin and dapagliflozin [61, 62, 63]. A recent multicenter observational study included 220 patients receiving SGLT2i therapy (mean age: 77 years; mean LVEF: 46%), who were compared to 220 propensity score–matched controls, and found that SGLT2i treatment was generally well tolerated (4.5% discontinuation rate) [64]. Over a median follow-up of 28 months, SGLT2i use was associated with reduced all-cause mortality (hazard ratio [HR] 0.57; 95% confidence interval [CI] 0.37–0.89; *p* = 0.010), cardiovascular mortality (HR 0.41; 95% CI 0.24–0.71; *p* < 0.001), HF hospitalizations (HR 0.57; 95% CI 0.36–0.91; *p* = 0.014), and the combined endpoint of cardiovascular death or HF hospitalization (HR 0.57; 95% CI 0.38–0.84; *p* = 0.003) [64]. Hence, based on existing data, SGLT2i therapy appears to be safe and effective for initiation in patients with ATTR-CA and HF, provided there are no contraindications—similar to those applicable in non-amyloid HF populations.

While the discussion of GDMT for HF in the context of ATTR-CA is clinically important, it is crucial to acknowledge that much of the available evidence is derived from observational studies, registries, or retrospective analyses. These studies, while valuable for hypothesis generation and real-world insight, are inherently limited by confounding factors and potential selection bias. For example, patients who are prescribed β-blockers or ACEi/ARBs may differ systematically from those who are not—in terms of disease severity, comorbidities, or physician preference—which can influence outcomes independent of the therapies themselves. Moreover, tolerability and prescription patterns may reflect underlying cardiac phenotype (e.g., degree of restrictive physiology or hypotension), further complicating causal inference. The lack of randomized controlled trials in this area limits the ability to draw definitive conclusions about efficacy or safety. Therefore, while observational data suggest possible benefit or even harm of specific GDMT components in ATTR-CA, these findings must be interpreted cautiously. A more nuanced understanding of patient selection and treatment response will require prospective studies or ideally randomized trials tailored to the ATTR-CA population.

#### 6.1.3 Management of Arrhythmias

Atrial fibrillation is the most common rhythm disturbance in ATTR-CA [65]. Rhythm control strategy is often preferred as these patients are often unable to tolerate rate control therapies [66]. Data on catheter ablation is still limited. In addition, ATTR-CA patients with atrial fibrillation are at a significantly higher risk of stroke than non-amyloid patients with atrial fibrillation [38]. Anticoagulation may be considered irrespective of CHA2DS2-VASc [38]. Direct oral anticoagulants are safe to use as an alternative to vitamin K antagonists [67]; however, caution is warranted in the presence of significant renal or hepatic impairment, which may influence drug metabolism and bleeding risk [68].

Bradyarrhythmias are more prevalent in ATTRwt-CA than ATTRv-CA [69]. Pacemaker implantation, when indicated, can help provide symptomatic relief but does not confer mortality benefit [69]. Progression of conduction system disease is common and often leads to increased right ventricular pacing burden with time. Therefore, cardiac resynchronizing therapy (biventricular therapy) is often considered a better and safer option in these patients [70]. The role of prophylactic pacemaker implantation is controversial.

Ventricular arrhythmias are common in ATTR-CA [71]. Data on the efficacy of implantable cardioverter-defibrillators (ICDs) for primary or secondary prevention in ATTR-CA are limited to small-scale observational studies. While universal ICD placement in all patients with ATTR-CA remains controversial, selected patients—particularly those with a history of sustained ventricular arrhythmias—may derive benefit, especially in the context of secondary prevention [72, 73, 74, 75, 76, 77, 78].

### 6.2 Amyloid-Specific Therapies

#### 6.2.1 TTR Silencers

In patients with ATTRv with polyneuropathy, several gene-silencing therapies have shown efficacy in randomized controlled trials. Eplontersen, in the NEUROTTRansform trial (n = 144), reduced serum TTR by 81.7%, stabilized neuropathy progression, and improved quality of life over 66 weeks (*p* < 0.001 for all) [79]. Inotersen, in the NEUROTTR trial (n = 172), also slowed neuropathy and preserved quality of life, but was associated with significant safety concerns including thrombocytopenia (>50%) and glomerulonephritis (~3%) [80]. Patisiran, in the APOLLO trial (n = 225), significantly improved neuropathy, autonomic symptoms, and cardiac biomarkers in a cardiac subgroup, with sustained TTR knockdown and favorable tolerability [81]. Vutrisiran, evaluated in HELIOSA (n = 164), showed comparable efficacy to patisiran with quarterly subcutaneous dosing [82], and in HELIOSB (n = 654), significantly reduced all-cause mortality and cardiovascular events in patients with ATTR-CA [83], confirming its therapeutic potential in both polyneuropathy and cardiomyopathy settings. Looking ahead, ongoing trials of eplontersen and next-generation silencers aim to expand treatment indications, improve cardiac outcomes, and offer more convenient or durable delivery options.

#### 6.2.2 TTR Stabilizers

Among TTR stabilizers, tafamidis remains the only widely approved agent based on robust phase III evidence. In the ATTR-ACT trial (n = 441), tafamidis significantly reduced all-cause mortality and cardiovascular hospitalizations over 30 months in patients with ATTR-CA, while also slowing functional decline and preserving quality of life [59]. Acoramidis, evaluated in the ATTRibute-CM trial (n = 632), demonstrated a significant benefit on a hierarchical composite outcome (mortality, cardiovascular hospitalizations, NT-proBNP, and 6-minute walk distance), with a favorable safety profile and a win ratio of 1.8 compared to placebo [84]. While not yet widely adopted, diflunisal, a nonsteroidal anti-inflammatory drug with TTR-stabilizing properties, has shown beneficial effects in small observational studies and early trials, particularly in slowing neuropathy progression in ATTRv with predominant neuropathy, though its use is limited by safety concerns (e.g., renal and gastrointestinal toxicity) and lack of large-scale randomized controlled trial (RCT) data. Looking ahead, newer stabilizers may offer enhanced binding and clinical benefit, and combination trials with silencers may shape future treatment paradigms [85].

#### 6.2.3 TTR Degraders

Emerging TTR degraders, which aim to target and remove existing amyloid fibrils rather than simply inhibiting their formation or production, represent a promising therapeutic avenue in ATTRCA. Among these, the combination of doxycycline—a tetracycline antibiotic—with tauroursodeoxycholic acid (TUDCA) has gained experimental traction. In animal models of ATTR, this duo effectively reduced both fibrillar and nonfibrillar deposits, and in a small phase II openlabel study, the combination stabilized cardiac and neurologic disease in most treated patients over one year with an acceptable safety profile [86]. Encouragingly, a larger randomized phase III trial comparing doxycycline/TUDCA plus supportive therapy versus supportive care alone (NCT03481972) is currently underway [87]. Meanwhile, a broader class of antiamyloid approaches is under active investigation, including monoclonal antibodies like PRX004 (now NNC6019) and ALXN2220 (formerly NI006), designed to directly bind and facilitate clearance of TTR deposits; both are being evaluated in Phase II/III trials in ATTRCA patients [87]. Taken together, these strategies offer hope for therapeutic removal of established amyloid, potentially complementing existing stabilizers and silencers in the management of ATTRCA.

### 6.3 Genetic Counseling and Family Screening

Despite growing momentum toward proactive family screening in ATTRv-CA, current discourse continues to underrepresent the critical roles of genetic counseling and cascade testing. This underrepresentation is significant, given the critical role of early identification in altering disease trajectory. Genetic counseling provides essential support by educating at-risk relatives about inheritance patterns, clinical implications, and testing options, while also guiding interpretation of variants. Family screening enables the detection of preclinical disease and timely intervention. A recent multinational evaluation of the 2021 ESC consensus recommendations underscores its importance: among 159 asymptomatic relatives across 10 European centers, 25% were already diagnosed with ATTRv-CA at baseline, and 13% of these had no red flag abnormalities on standard ECG, echocardiography, or biomarkers [88]. Additionally, 9.4% developed ATTRv-CA during follow-up, demonstrating the value of serial screening. The ESC criteria showed a high negative predictive value (97%), supporting their clinical utility, while male sex and rare TTR variants were associated with greater disease risk. These findings reinforce the need for structured genetic counseling and systematic screening protocols to ensure early diagnosis and enable timely therapeutic intervention—particularly important in an era of emerging disease-modifying treatments.

## 7. Conclusions

ATTR-CA has emerged as a significant and underrecognized contributor to HF, affecting both HFpEF and HFrEF populations. Advances in imaging and greater clinical awareness have revealed a higher community prevalence than previously appreciated, with ATTR-CA often mimicking other cardiac conditions and evading timely diagnosis. While conventional GDMT for HF offers symptomatic and mortality benefits, these agents have traditionally been thought to be poorly tolerated in ATTR-CA due to the low, fixed stroke volume and autonomic dysfunction. Recent observational data, however, suggest that some patients—particularly those with systolic dysfunction—can tolerate and potentially benefit from beta-blockers, ACEi/ARBs, and MRAs with careful monitoring. Most importantly, amyloid-specific therapies have revolutionized the management of ATTR-CA. Stabilizers such as tafamidis have demonstrated robust mortality and morbidity benefits, while gene silencers offer additional disease-modifying potential. As therapeutic options expand, early recognition and subtype identification will be critical to optimizing outcomes. Broader screening strategies and continued research into tailored treatment approaches will be essential in improving the care of patients with this complex and increasingly treatable form of HF.

## References

[1] Gillmore JD, Maurer MS, Falk RH, Merlini G, Damy T, Dispenzieri A (2016). Nonbiopsy Diagnosis of Cardiac Transthyretin Amyloidosis. *Circulation*.

[2] Masri A, Bukhari S, Eisele YS, Soman P (2020). Molecular Imaging of Cardiac Amyloidosis. *Journal of Nuclear Medicine: Official Publication, Society of Nuclear Medicine*.

[3] Alonso M, Neicheril RK, Manla Y, McDonald ML, Sanchez A, Lafave G (2024). Transthyretin cardiac amyloid: Broad heart failure phenotypic spectrum and implications for diagnosis. *ESC Heart Failure*.

[4] García-Pavía P, García-Pinilla JM, Lozano-Bahamonde A, Yun S, García-Quintana A, Gavira-Gómez JJ (2025). Prevalence of transthyretin cardiac amyloidosis in patients with heart failure with preserved ejection fraction: the PRACTICA study.

[5] Ruiz-Hueso R, Salamanca-Bautista P, Quesada-Simón MA, Yun S, Conde-Martel A, Morales-Rull JL (2023). Estimating the Prevalence of Cardiac Amyloidosis in Old Patients with Heart Failure-Barriers and Opportunities for Improvement: The PREVAMIC Study. *Journal of Clinical Medicine*.

[6] Achten A, van Empel VPM, Weerts J, Mourmans S, Brunner-La Rocca HP, Sanders-van Wijk S (2025). Prevalence of transthyretin amyloid cardiomyopathy in an unselected cohort with heart failure with preserved ejection fraction. *Netherlands Heart Journal: Monthly Journal of the Netherlands Society of Cardiology and the Netherlands Heart Foundation*.

[7] AbouEzzeddine OF, Davies DR, Scott CG, Fayyaz AU, Askew JW, McKie PM (2021). Prevalence of Transthyretin Amyloid Cardiomyopathy in Heart Failure With Preserved Ejection Fraction. *JAMA Cardiology*.

[8] Lindmark K, Pilebro B, Sundström T, Lindqvist P (2021). Prevalence of wild type transtyrethin cardiac amyloidosis in a heart failure clinic. *ESC Heart Failure*.

[9] González-López E, Gallego-Delgado M, Guzzo-Merello G, de Haro-Del Moral FJ, Cobo-Marcos M, Robles C (2015). Wild-type transthyretin amyloidosis as a cause of heart failure with preserved ejection fraction. *European Heart Journal*.

[10] Arruda-Olson AM, Zeldenrust SR, Dispenzieri A, Gertz MA, Miller FA, Bielinski SJ (2013). Genotype, echocardiography, and survival in familial transthyretin amyloidosis. *Amyloid: the International Journal of Experimental and Clinical Investigation: the Official Journal of the International Society of Amyloidosis*.

[11] Bashir Z, Younus A, Dhillon S, Kasi A, Bukhari S (2024). Epidemiology, diagnosis, and management of cardiac amyloidosis. *Journal of Investigative Medicine: the Official Publication of the American Federation for Clinical Research*.

[12] Reilly MM, Staunton H, Harding AE (1995). Familial amyloid polyneuropathy (TTR ala 60) in north west Ireland: a clinical, genetic, and epidemiological study. *Journal of Neurology, Neurosurgery, and Psychiatry*.

[13] Hewitt K, Starr N, Togher Z, Sulong S, Morris JP, Alexander M (2024). Spectrum of hereditary transthyretin amyloidosis due to T60A(p.Thr80Ala) variant in an Irish Amyloidosis Network. *Open Heart*.

[14] Bukhari S, Bashir Z, Shpilsky D, Eisele YS, Soman P (2020). Reduced ejection fraction at diagnosis is an independent predictor of mortality in transthyretin amyloid cardiomyopathy. *Circulation*.

[15] See ASY, Ho JSY, Chan MY, Lim YC, Yeo TC, Chai P (2022). Prevalence and Risk Factors of Cardiac Amyloidosis in Heart Failure: A Systematic Review and Meta-Analysis. *Heart, Lung & Circulation*.

[16] Goland S, Volodarsky I, Fabricant Y, Livschitz S, Tshori S, Cuciuc V (2021). Wild-type TTR amyloidosis among patients with unexplained heart failure and systolic LV dysfunction. *PloS One*.

[17] Bukhari S (2023). Cardiac amyloidosis: state-of-the-art review. *Journal of Geriatric Cardiology: JGC*.

[18] Bukhari S, Oliveros E, Parekh H, Farmakis D (2023). Epidemiology, Mechanisms, and Management of Atrial Fibrillation in Cardiac Amyloidosis. *Current Problems in Cardiology*.

[19] Giancaterino S, Urey MA, Darden D, Hsu JC (2020). Management of Arrhythmias in Cardiac Amyloidosis. *JACC. Clinical Electrophysiology*.

[20] Ruberg FL, Maurer MS (2024). Cardiac Amyloidosis Due to Transthyretin Protein: A Review. *JAMA*.

[21] Shetty NS, Pampana A, Patel N, Maurer MS, Goyal P, Li P (2024). Carpal Tunnel Syndrome and Transthyretin Amyloidosis in the All of Us Research Program. *Mayo Clinic Proceedings*.

[22] Sperry BW, Reyes BA, Ikram A, Donnelly JP, Phelan D, Jaber WA (2018). Tenosynovial and Cardiac Amyloidosis in Patients Undergoing Carpal Tunnel Release. *Journal of the American College of Cardiology*.

[23] Sperry BW, Khedraki R, Gabrovsek A, Donnelly JP, Kilpatrick S, Shapiro D (2021). Cardiac Amyloidosis Screening at Trigger Finger Release Surgery. *The American Journal of Cardiology*.

[24] Geller HI, Singh A, Alexander KM, Mirto TM, Falk RH (2017). Association Between Ruptured Distal Biceps Tendon and Wild-Type Transthyretin Cardiac Amyloidosis. *JAMA*.

[25] Eldhagen P, Berg S, Lund LH, Sörensson P, Suhr OB, Westermark P (2021). Transthyretin amyloid deposits in lumbar spinal stenosis and assessment of signs of systemic amyloidosis. *Journal of Internal Medicine*.

[26] Maurer MS, Smiley D, Simsolo E, Remotti F, Bustamante A, Teruya S (2022). Analysis of lumbar spine stenosis specimens for identification of amyloid. *Journal of the American Geriatrics Society*.

[27] Noory N, Westin O, Fosbøl E, Maurer MS, Gustafsson F (2024). Value of troponin and NT-proBNP to screen for cardiac amyloidosis after carpal tunnel syndrome surgery. *International Journal of Cardiology*.

[28] Grogan M, Scott CG, Kyle RA, Zeldenrust SR, Gertz MA, Lin G (2016). Natural History of Wild-Type Transthyretin Cardiac Amyloidosis and Risk Stratification Using a Novel Staging System. *Journal of the American College of Cardiology*.

[29] Zhelev Z, Ohtake H, Iwata M, Terasawa T, Rogers M, Peters JL (2019). Diagnostic accuracy of contemporary and high-sensitivity cardiac troponin assays used in serial testing, versus single-sample testing as a comparator, to triage patients suspected of acute non-ST-segment elevation myocardial infarction: a systematic review protocol. *BMJ Open*.

[30] Garcia-Pavia P, Bengel F, Brito D, Damy T, Duca F, Dorbala S (2021). Expert consensus on the monitoring of transthyretin amyloid cardiomyopathy. *European Journal of Heart Failure*.

[31] Freda BJ, Tang WHW, Van Lente F, Peacock WF, Francis GS (2002). Cardiac troponins in renal insufficiency: review and clinical implications. *Journal of the American College of Cardiology*.

[32] McKie PM, Burnett JC (2016). NT-proBNP: The Gold Standard Biomarker in Heart Failure. *Journal of the American College of Cardiology*.

[33] Gillmore JD, Damy T, Fontana M, Hutchinson M, Lachmann HJ, Martinez-Naharro A (2018). A new staging system for cardiac transthyretin amyloidosis. *European Heart Journal*.

[34] Rørth R, Jhund PS, Yilmaz MB, Kristensen SL, Welsh P, Desai AS (2020). Comparison of BNP and NT-proBNP in Patients With Heart Failure and Reduced Ejection Fraction. *Circulation. Heart Failure*.

[35] Cipriani A, De Michieli L, Porcari A, Licchelli L, Sinigiani G, Tini G (2022). Low QRS Voltages in Cardiac Amyloidosis: Clinical Correlates and Prognostic Value. *JACC. CardioOncology*.

[36] Olausson E, Wertz J, Fridman Y, Bering P, Maanja M, Niklasson L (2023). Diffuse myocardial fibrosis associates with incident ventricular arrhythmia in implantable cardioverter defibrillator recipients. *medRxiv*.

[37] Martini N, Sinigiani G, De Michieli L, Mussinelli R, Perazzolo Marra M, Iliceto S (2024). Electrocardiographic features and rhythm disorders in cardiac amyloidosis. *Trends in Cardiovascular Medicine*.

[38] Bukhari S, Barakat AF, Eisele YS, Nieves R, Jain S, Saba S (2021). Prevalence of Atrial Fibrillation and Thromboembolic Risk in Wild-Type Transthyretin Amyloid Cardiomyopathy. *Circulation*.

[39] Bukhari S, Bashir Z (2024). Diagnostic Modalities in the Detection of Cardiac Amyloidosis. *Journal of Clinical Medicine*.

[40] Muller SA, Achten A, van der Meer MG, Zwetsloot PP, Sanders-van Wijk S, van der Harst P (2024). Absence of an increased wall thickness does not rule out cardiac amyloidosis. *Amyloid: the International Journal of Experimental and Clinical Investigation: the Official Journal of the International Society of Amyloidosis*.

[41] Bashir Z, Musharraf M, Azam R, Bukhari S (2024). Imaging modalities in cardiac amyloidosis. *Current Problems in Cardiology*.

[42] Cuddy SAM, Chetrit M, Jankowski M, Desai M, Falk RH, Weiner RB (2022). Practical Points for Echocardiography in Cardiac Amyloidosis. *Journal of the American Society of Echocardiography: Official Publication of the American Society of Echocardiography*.

[43] Virkud AV, Chang PP, Funk MJ, Kshirsagar AV, Edwards JK, Pate V (2024). Comparative Effect of Loop Diuretic Prescription on Mortality and Heart Failure Readmission. *The American Journal of Cardiology*.

[44] Rapezzi C, Aimo A, Barison A, Emdin M, Porcari A, Linhart A (2022). Restrictive cardiomyopathy: definition and diagnosis. *European Heart Journal*.

[45] Rosenberg J, Gustafsson F, Galatius S, Hildebrandt PR (2005). Combination therapy with metolazone and loop diuretics in outpatients with refractory heart failure: an observational study and review of the literature. *Cardiovascular Drugs and Therapy*.

[46] Cheng RK, Levy WC, Vasbinder A, Teruya S, De Los Santos J, Leedy D (2020). Diuretic Dose and NYHA Functional Class Are Independent Predictors of Mortality in Patients With Transthyretin Cardiac Amyloidosis. *JACC. CardioOncology*.

[47] (1999). The Cardiac Insufficiency Bisoprolol Study II (CIBIS-II): a randomised trial. *Lancet (London, England)*.

[48] (1999). Effect of metoprolol CR/XL in chronic heart failure: Metoprolol CR/XL Randomised Intervention Trial in Congestive Heart Failure (MERIT-HF). *Lancet (London, England)*.

[49] Oghina S, Bougouin W, Bézard M, Kharoubi M, Komajda M, Cohen-Solal A (2021). The Impact of Patients With Cardiac Amyloidosis in HFpEF Trials. *JACC. Heart Failure*.

[50] Packer M, Coats AJ, Fowler MB, Katus HA, Krum H, Mohacsi P (2001). Effect of carvedilol on survival in severe chronic heart failure. *The New England Journal of Medicine*.

[51] Yusuf S, Pitt B, Davis CE, Hood WB, Cohn JN, SOLVD Investigators (1991). Effect of enalapril on survival in patients with reduced left ventricular ejection fractions and congestive heart failure. *The New England Journal of Medicine*.

[52] Solomon SD, Wang D, Finn P, Skali H, Zornoff L, McMurray JJV (2004). Effect of candesartan on cause-specific mortality in heart failure patients: the Candesartan in Heart failure Assessment of Reduction in Mortality and morbidity (CHARM) program. *Circulation*.

[53] Pitt B, Zannad F, Remme WJ, Cody R, Castaigne A, Perez A (1999). The effect of spironolactone on morbidity and mortality in patients with severe heart failure. Randomized Aldactone Evaluation Study Investigators. *The New England Journal of Medicine*.

[54] Zannad F, McMurray JJV, Krum H, van Veldhuisen DJ, Swedberg K, Shi H (2011). Eplerenone in patients with systolic heart failure and mild symptoms. *The New England Journal of Medicine*.

[55] Cleland JGF, Bunting KV, Flather MD, Altman DG, Holmes J, Coats AJS (2018). Beta-blockers for heart failure with reduced, mid-range, and preserved ejection fraction: an individual patient-level analysis of double-blind randomized trials. *European Heart Journal*.

[56] Aimo A, Vergaro G, Castiglione V, Rapezzi C, Emdin M (2020). Safety and Tolerability of Neurohormonal Antagonism in Cardiac Amyloidosis. *European Journal of Internal Medicine*.

[57] Tini G, Cappelli F, Biagini E, Musumeci B, Merlo M, Crotti L (2021). Current patterns of beta-blocker prescription in cardiac amyloidosis: an Italian nationwide survey. *ESC Heart Failure*.

[58] Garcia-Pavia P, Rapezzi C, Adler Y, Arad M, Basso C, Brucato A (2021). Diagnosis and treatment of cardiac amyloidosis. A position statement of the European Society of Cardiology Working Group on Myocardial and Pericardial Diseases. *European Journal of Heart Failure*.

[59] Maurer MS, Schwartz JH, Gundapaneni B, Elliott PM, Merlini G, Waddington-Cruz M (2018). Tafamidis Treatment for Patients with Transthyretin Amyloid Cardiomyopathy. *The New England Journal of Medicine*.

[60] Ioannou A, Massa P, Patel RK, Razvi Y, Porcari A, Rauf MU (2023). Conventional heart failure therapy in cardiac ATTR amyloidosis. *European Heart Journal*.

[61] Anker SD, Butler J, Filippatos G, Ferreira JP, Bocchi E, Böhm M (2021). Empagliflozin in Heart Failure with a Preserved Ejection Fraction. *The New England Journal of Medicine*.

[62] McMurray JJV, Solomon SD, Inzucchi SE, Køber L, Kosiborod MN, Martinez FA (2019). Dapagliflozin in Patients with Heart Failure and Reduced Ejection Fraction. *The New England Journal of Medicine*.

[63] Solomon SD, McMurray JJ, Claggett B, de Boer RA, DeMets D, Hernandez AF (2022). Dapagliflozin in heart failure with mildly reduced or preserved ejection fraction. *New England Journal of Medicine*.

[64] Porcari A, Cappelli F, Nitsche C, Tomasoni D, Sinigiani G, Longhi S (2024). SGLT2 Inhibitor Therapy in Patients With Transthyretin Amyloid Cardiomyopathy. *Journal of the American College of Cardiology*.

[65] Hartnett J, Jaber W, Maurer M, Sperry B, Hanna M, Collier P (2021). Electrophysiological Manifestations of Cardiac Amyloidosis: JACC: CardioOncology State-of-the-Art Review. *JACC. CardioOncology*.

[66] Bukhari S, Khan SZ, Bashir Z (2023). Atrial Fibrillation, Thromboembolic Risk, and Anticoagulation in Cardiac Amyloidosis: A Review. *Journal of Cardiac Failure*.

[67] Mitrani LR, De Los Santos J, Driggin E, Kogan R, Helmke S, Goldsmith J (2021). Anticoagulation with warfarin compared to novel oral anticoagulants for atrial fibrillation in adults with transthyretin cardiac amyloidosis: comparison of thromboembolic events and major bleeding. *Amyloid: the International Journal of Experimental and Clinical Investigation: the Official Journal of the International Society of Amyloidosis*.

[68] Bukhari S, Ghoweba M, Khan SZ, Saati A, Gomes M (2025). Direct oral anticoagulants: Challenging prescribing scenarios in everyday practice. *Cleveland Clinic Journal of Medicine*.

[69] Garcia-Pavia P, Gonzalez-Lopez E, Anderson LJ, Cappelli F, Damy T, Fontana M (2025). Non-amyloid specific treatment for transthyretin cardiac amyloidosis: a clinical consensus statement of the ESC Heart Failure Association. *European Heart Journal*.

[70] Bukhari S, Khan SZ, Ghoweba M, Khan B, Bashir Z (2024). Arrhythmias and Device Therapies in Cardiac Amyloidosis. *Journal of Clinical Medicine*.

[71] Bukhari S, Khan B (2023). Prevalence of ventricular arrhythmias and role of implantable cardioverter-defibrillator in cardiac amyloidosis. *Journal of Cardiology*.

[72] Kristen AV, Dengler TJ, Hegenbart U, Schonland SO, Goldschmidt H, Sack FU (2008). Prophylactic implantation of cardioverter-defibrillator in patients with severe cardiac amyloidosis and high risk for sudden cardiac death. *Heart Rhythm*.

[73] Varr BC, Zarafshar S, Coakley T, Liedtke M, Lafayette RA, Arai S (2014). Implantable cardioverter-defibrillator placement in patients with cardiac amyloidosis. *Heart Rhythm*.

[74] Hamon D, Algalarrondo V, Gandjbakhch E, Extramiana F, Marijon E, Elbaz N (2016). Outcome and incidence of appropriate implantable cardioverter-defibrillator therapy in patients with cardiac amyloidosis. *International Journal of Cardiology*.

[75] Lin G, Dispenzieri A, Kyle R, Grogan M, Brady PA (2013). Implantable cardioverter defibrillators in patients with cardiac amyloidosis. *Journal of Cardiovascular Electrophysiology*.

[76] Higgins AY, Annapureddy AR, Wang Y, Minges KE, Lampert R, Rosenfeld LE (2020). Survival Following Implantable Cardioverter-Defibrillator Implantation in Patients With Amyloid Cardiomyopathy. *Journal of the American Heart Association*.

[77] Donnellan E, Wazni OM, Hanna M, Saliba W, Jaber W, Kanj M (2020). Primary prevention implantable cardioverter-defibrillators in transthyretin cardiac amyloidosis. *Pacing and Clinical Electrophysiology: PACE*.

[78] Brown MT, Yalamanchili S, Evans ST, Ram P, Blank EA, Lyle MA (2022). Ventricular arrhythmia burden and implantable cardioverter-defibrillator outcomes in transthyretin cardiac amyloidosis. *Pacing and Clinical Electrophysiology: PACE*.

[79] Coelho T, Marques W, Dasgupta NR, Chao CC, Parman Y, França MC (2023). Eplontersen for Hereditary Transthyretin Amyloidosis With Polyneuropathy. *JAMA*.

[80] Benson MD, Waddington-Cruz M, Berk JL, Polydefkis M, Dyck PJ, Wang AK (2018). Inotersen Treatment for Patients with Hereditary Transthyretin Amyloidosis. *The New England Journal of Medicine*.

[81] Adams D, Gonzalez-Duarte A, O’Riordan WD, Yang CC, Ueda M, Kristen AV (2018). Patisiran, an RNAi Therapeutic, for Hereditary Transthyretin Amyloidosis. *The New England Journal of Medicine*.

[82] Adams D, Tournev IL, Taylor MS, Coelho T, Planté-Bordeneuve V, Berk JL (2023). Efficacy and safety of vutrisiran for patients with hereditary transthyretin-mediated amyloidosis with polyneuropathy: a randomized clinical trial. *Amyloid: the International Journal of Experimental and Clinical Investigation: the Official Journal of the International Society of Amyloidosis*.

[83] Fontana M, Berk JL, Gillmore JD, Witteles RM, Grogan M, Drachman B (2025). Vutrisiran in Patients with Transthyretin Amyloidosis with Cardiomyopathy. *The New England Journal of Medicine*.

[84] Gillmore JD, Judge DP, Cappelli F, Fontana M, Garcia-Pavia P, Gibbs S (2024). Efficacy and Safety of Acoramidis in Transthyretin Amyloid Cardiomyopathy. *The New England Journal of Medicine*.

[85] Fontana M, Aimo A, Emdin M, Porcari A, Solomon SD, Hawkins PN (2025). Transthyretin amyloid cardiomyopathy: from cause to novel treatments. *European Heart Journal*.

[86] Obici L, Cortese A, Lozza A, Lucchetti J, Gobbi M, Palladini G (2012). Doxycycline plus tauroursodeoxycholic acid for transthyretin amyloidosis: a phase II study. *Amyloid: the International Journal of Experimental and Clinical Investigation: the Official Journal of the International Society of Amyloidosis*.

[87] Hellenbart EL, Ipema HJ, Rodriguez-Ziccardi MC, Krishna H, DiDomenico RJ (2025). Disease-modifying therapies for amyloid transthyretin cardiomyopathy: Current and emerging medications. *Pharmacotherapy*.

[88] Muller SA, Peiró-Aventin B, Biagioni G, Tini G, Saturi G, Kronberger C (2024). Evaluation of the 2021 ESC recommendations for family screening in hereditary transthyretin cardiac amyloidosis. *European Journal of Heart Failure*.

